# Supplementary material for: Strategies to Improve Vaccination among At-Risk Adults and the Elderly in Italy
Source: Vaccines (Basel). 2020 Jul 4;8(3):358. doi: 10.3390/vaccines8030358 (PMC7565655; doi:10.3390/vaccines8030358)
Supplement: Supplementary file 1 [file vaccines-08-00358-s001.pdf]

BRAVE PROJECT SURVEY

Best practices improving Vaccination coverage among at-risk adults and Elderly.

SEZIONE 1: DATI PROFESSIONALI

Nella prima sezione del questionario vengono richieste informazioni generali circa la Sua professione e sede lavorativa, sull'adozione delle raccomandazioni dell'attuale PNPV e dell'anagrafe vaccinale da parte della Sua Regione.

1. POSIZIONE RICOPERTA ……………………………………………………………………

2. REGIONE ……………………………………………………………………………………

3. PROVINCIA ……………………………………………………………………………

4. STRUTTURA DI AFFERENZA ………………………………………………………………

5. Nella Sua Regione sono state recepite le indicazioni del PNPV 2017-2019?
   5.1 VACCINAZIONE ANTINFLUENZALE
      - o Sì
      - o NO
      - o PARZIALMENTE

   5.2 VACCINAZIONE ANTI-PNEUMOCOCCO
      - o Sì
      - o NO
      - o PARZIALMENTE

   5.3 VACCINAZIONE ANTI-HERPES ZOSTER
      - o Sì
      - o NO
      - o PARZIALMENTE

6. Nella Sua Regione, è stata implementata l'anagrafe vaccinale informatizzata?
   - o SI
   - o NO

SEZIONE 1 - RISPOSTE

	1.	2.	3.	4.	5.1	5.2	5.3	6.
A	Direttore UOC	Lombardia	Mantova Cremona	Dipartimento Prevenzione	SI	SI	SI	SI
B	Dirigente medico medicina preventiva	Calabria	Crotone	Dipartimento di prevenzione	SI	SI	SI	SI
C	Responsabile Medico Centro Vaccinale	Calabria	Cosenza	U.O.C. Igiene Pubblica Medicina Preventiva Dipartimento di Prevenzione	SI	SI	SI	SI
D	Dirigente medico	Lombardia	Milano	UO Prevenzione DG Welfare	SI	SI	SI	SI
E	Direzione Generale Tutela della Salute e coordinamento del SSR UOD 17	Campania	Napoli	UOD 17 Attività Consultoriali e Assistenza Materno Infantile	SI	SI	SI	SI
F	Responsabile servizio igiene e sanità pubblica	Friuli Venezia Giulia	Pordenone	Dipartimento di Prevenzione	SI	SI	SI	SI
G	Professore associato	Puglia	Bari	DIMO UNIBA	SI	SI	SI	SI
H	Dirigente Medico	Marche	Ancona	SISP	SI	SI	SI	SI
I	Dirigente medico	Liguria	Genova	S.C. Prevenzione A.Li.Sa.	SI	SI	SI	SI
J	Direttore di dipartimento di prevenzione	Puglia	Taranto	Dipartimento di prevenzione	SI	SI	SI	SI
K	Referente UOC Epidemiologia e Prevenzione	Campania	Napoli	ASL Napoli2 Nord-Dipartimento di Prevenzione	SI	SI	SI	SI
L	Dirigente medico	Liguria	Imperia	Igiene e sanità pubblica Ventimiglia	SI	SI	SI	SI
M	Infermiera	Liguria	Imperia	Distretto Ventimiglia	SI	SI	SI	SI
N	Direttore	Lombardia	Sondrio	ATS Montagna - Dipartimento Igiene e Prevenzione Sanitaria	SI	SI	SI	SI
O	Dirigente	Piemonte	Piemonte	SEREMI-ASL AL	SI	SI	SI	SI
P	Dirigente Medico	Trentino-Alto Adige	Bolzano	Azienda Sanitaria dell'Alto Adige, SISP Merano	PARZIAL-MENTE	PARZIAL-MENTE	SI	SI
Q	Dirigente medico	Lazio	Latina	SISP ASL Latina	SI	SI	SI	SI
R	Referente regionale malattie infettive e vaccinazioni	Puglia	Foggia	Università di Foggia	SI	SI	SI	SI
S	Referente regionale malattie infettive e vaccinazioni	Puglia	Foggia	Università di Foggia	SI	SI	SI	SI

SEZIONE 2: INDAGINE INIZIATIVE VACCINALI

Di seguito vengono proposti tre questionari, da completare fornendo informazioni sulle vaccinazioni oggetto di approfondimento (vaccinazione antinfluenzale, anti-pneumococcica e anti-Herpes Zoster) e rivolte alla popolazione adulta a rischio (con comorbosità) e anziana.

Per ciascuna vaccinazione è prevista la compilazione di schede concernenti eventuali iniziative/strategie innovative – riferite alla vaccinazione in contesti non convenzionali e/o attraverso modelli alternativi a quelli abitualmente adottati – messe in atto nella Sua Regione.

<u>VACCINAZIONE ANTINFLUENZALE</u>

1. Riguardo la vaccinazione **ANTINFLUENZALE** per gli anziani (età ≥65 anni) e pazienti affetti da condizioni a rischio, è a conoscenza di iniziative/strategie vaccinali organizzate negli ultimi 5 anni al di fuori degli ambulatori vaccinali e degli ambulatori di singoli Medici di Medicina Generale (MMG) nella sua Regione?
    - SI
    - SI, PIÙ DI UNA DI DIVERSO TIPO (specificare e compilare, per ciascuna iniziativa/strategia, l'apposita scheda)
    - NO

<u>VACCINAZIONE ANTI-HERPES ZOSTER VIRUS</u>

1. Riguardo la vaccinazione **ANTI-HERPES ZOSTER VIRUS** per gli anziani (età ≥65 anni) e pazienti affetti da condizioni a rischio, è a conoscenza di iniziative/strategie vaccinali organizzate negli ultimi 5 anni al di fuori degli ambulatori vaccinali e degli ambulatori di singoli Medici di Medicina Generale (MMG) nella sua Regione?
    - SI
    - SI, PIÙ DI UNA DI DIVERSO TIPO (specificare e compilare, per ciascuna iniziativa/strategia, l'apposita scheda)
    - NO

<u>VACCINAZIONE ANTIPNEUMOCOCCICA</u>

1. Riguardo la vaccinazione **ANTIPNEUMOCOCCICA** per gli anziani (età ≥65 anni) e pazienti affetti da condizioni a rischio, è a conoscenza di iniziative/strategie vaccinali organizzate negli ultimi 5 anni al di fuori degli ambulatori vaccinali e degli ambulatori di singoli Medici di Medicina Generale (MMG) nella sua Regione?
    - SI
    - SI, PIÙ DI UNA DI DIVERSO TIPO (specificare e compilare, per ciascuna iniziativa/strategia, l'apposita scheda)
    - NO

SEZIONE 2 – RISPOSTE

	1. ANTINFLUENZALE	1. ANTI-PNEUMOCOCCICA	1. ANTI-HERPES ZOSTER
**A**	Si, Una	Si, Una	No
**B**	Si, Una	No	No
**C**	Si, Una	No	No
**D**	Si, Una	Si, Una	No
**E**	No	No	No
**F**	Si, più di una di diverso tipo	No	No
**G**	Si, Una	No	No
**H**	Si, più di una di diverso tipo	No	No
**I**	No	No	No
**J**	No	No	No
**K**	No	No	No
**L**	No	No	No
**M**	No	No	No
**N**	Si, Una	Si, Una	Si, Una
**O**	No	No	No
**P**	Si, Una	Si, Una	No
**Q**	Si, Una	No	No
**R**	No	No	No
**S**	Si, più di una di diverso tipo	Si, più di una di diverso tipo	No

SEZIONE 3: STRATEGIE/INIZIATIVE

Questa sezione è rivolta a raccogliere informazioni organizzative sulle eventuali iniziative da Lei precedentemente inserite.

Per ogni iniziativa Le chiediamo, gentilmente, di compilare la scheda preposta (può anche condividere, ove possibile, link ed allegati relativi alle iniziative proposte ed eventuali contatti di un referente direttamente coinvolto nelle stesse).

Il limite massimo di schede compilabile è tre, pertanto in caso di un numero superiore, Le chiediamo, cortesemente, di compilare la sezione finale (testo libero) seguendo la stessa impostazione delle schede proposte di seguito ed allegando, ove possibile, il relativo materiale a disposizione (link, documentazione specifica, delibere regionali, contatti referente iniziativa ecc).

STRATEGIE/INIZIATIVE n. 1, 2, 3…- VACCINAZIONE ANTINFLUENZALE/VACCINAZIONE ANTI-HERPES ZOSTER VIRUS/ VACCINAZIONE ANTIPNEUMOCOCCICA

1. A quale popolazione target era/è rivolta l'iniziativa (più di una risposta possibile)?
   - ANZIANI DI ETÀ ≥65 ANNI

- ADULTI CON CONDIZIONI A RISCHIO (es. malattie cardiologiche o polmonari croniche, diabetici)
- NON SO
- Altro

  ……………………………………………………………………………………….

2. Nel caso in cui la popolazione target siano adulti con condizione a rischio, potrebbe indicare quale?

3. Indichi il setting in cui queste iniziative/strategie vaccinali sono state/sono organizzate *(più di una risposta possibile)*:
   - POSTAZIONI VACCINALI MOBILI
   - OSPEDALE – ALL'INTERNO DEI REPARTI DI DEGENZA
   - OSPEDALE – AMBULATORIO
   - RESIDENZE SANITARIE ASSISTENZIALI (RSA O RSSA)
   - CENTRO DIABETOLOGICO
   - FARMACIE
   - CASE DI RECLUSIONE/CIRCONDARIALI
   - CASE DI RIPOSO
   - AMBIENTI LAVORATIVI (SCUOLE, AZIENDE PUBBLICHE/PRIVATE, ECC.)
   - NON SO
   - ALTRO (Specificare ……………………………………………………………………………)

4. Nel caso in cui il setting sia l'ospedale, potrebbe indicare quale reparto/ambulatorio (anche più di uno)?

5. Quali professionisti sanitari erano/sono coinvolti nell'iniziativa/strategia vaccinale indicata?
   - MEDICO
   - INFERMIERE
   - Altro

     ……………………………………………………………………………………

6. Se nell'ambito dell'iniziativa/strategia l'offerta vaccinale avveniva/avviene tramite:
   - ACCESSO SPONTANEO AL SERVIZIO SU RICHIESTA DELLA POPOLAZIONE TARGET
     - Con prenotazione cartacea
     - Con prenotazione telefonica
     - Con prenotazione telematica

     Per ognuna, quanto era il tempo di attesa?
       - 0-14gg
       - 15-30 gg

        ▪ Oltre 30 gg

- CHIAMATA ATTIVA FINALIZZATA SOLO ALL'INFORMAZIONE DELL'INIZIATIVA VACCINALE, con quale modalità avviene la chiamata?
    - Con lettera di invito
    - Tramite contatto telefonico
    - Tramite sms
    - Tramite email
    - Altro: ……………………………………………………………………………………………
- CHIAMATA ATTIVA CON APPUNTAMENTO PROGRAMMATO PER LA VACCINAZIONE, con quale modalità avviene la chiamata?
    - Con lettera di invito
    - Tramite contatto telefonico
    - Tramite sms
    - Tramite email
    - Altro ……………………………………………………………………………………………

7. È stato/è previsto un incontro con un operatore sanitario che spiegasse/spieghi benefici e rischi della vaccinazione alla popolazione target (Counselling Vaccinale) prima della somministrazione del vaccino?
    - SI
    - NO

   7.1 In caso affermativo, chi ha effettuato/effettua questo incontro? …………………………………………

   7.2 In caso affermativo, è stato prodotto del materiale informativo di supporto al counselling?
    - SI
    - NO

8. La vaccinazione viene registrata nell'anagrafe vaccinale informatizzata?
    - SI
    - NO

9. È stata/É prevista una valutazione dell'iniziativa/strategia vaccinale?
    - SI
    - NO
    - NON SO

   9.1 In caso affermativo, quali sono stati gli aspetti indagati?
    - PERCENTUALE DI ADESIONE DELLA POPOLAZIONE TARGET INVITATA
    - COPERTURA VACCINALE
    - COSTI

- GRADIMENTO DA PARTE DELLA POPOLAZIONE TARGET
- ALTRO: ……………………………………………………………………………………

9.2 In caso affermativo, i risultati della valutazione sono stati resi pubblici?

- SI
- NO

10. Può fornire uno o più nominativi con relativi recapiti dei responsabili dell'iniziativa/strategia vaccinale, da contattare per ulteriori approfondimenti? ……………………………………………………………

11. Qualora disponibili, può fornire link e allegati relativi alla strategia/iniziativa descritta? ………………………………………………………………………………………… …………………

12. È a conoscenza di altre strategie/iniziative relative alla vaccinazione antinfluenzale?
    - SI
    - NO

SEZIONE 3- RISPOSTE

STRATEGIA/INIZIATIVA n. 1- VACCINAZIONE ANTINFLUENZALE

	1	2	3	4	5	6.1 [Con prenotazione cartacea]	6.1 [Con prenotazione telefonica]	6.1 [Con prenotazione telematica]	6.2	6.3	7	7.1	7.2	8	9	9.1	9.2	11	12
**A**	Anziani di età>65 anni; adulti con condizioni a rischio		Presa in carico		Gestori presa in carico										SI				No
**B**	Anziani di età>65 anni; Adulti con condizioni a rischio	Cardiopatici e broncopatici	Ospedale-ambulatorio; Residenze sanitarie assistenziali (RSA o RSSA); Farmacie; Case di reclusione / circondariali; Case di riposo	ambulatorio medico competente	Medico; infermiere	0-14gg			Con lettera di invito	Con lettera di invito	No		No	No	No				No
**C**	Anziani di età>65 anni; Adulti con condizioni a rischio		Ospedale - ambulatorio; Residenze sanitarie assistenziali (RSA o RSSA); Farmacie;		Medico; Infermiere	0-14gg	0-14gg	0-14gg	Con lettera di invito; Tramite sms	Con lettera di invito; Tramite sms	Si	Medico	Si	Si	Si	Percentuale adesione popolazione target invitata; Copertura vaccinale; Gradimento popolazione target	Si		Si

			Case di reclusione / circondariali; Case di riposo; Parrocchia																
D	Anziani di età>65 anni; Adulti con condizioni a rischio	Patologie comprese nel PNPV	Ospedale-all'interno dei reparti di degenza; Ospedale-ambulatorio; Residenze sanitarie assistenziali (RSA o RSSA); Case di riposo		Medico; Infermiere				Mezzi di comunicazione manifesti sito web		Si	Medici/ infermieri	Si	Si	Si	Percentuale adesione popolazione target invitata; Copertura vaccinale; Costi	No		No
E													SI						
F	Anziani di età>65 anni; Adulti con condizioni a rischio	Malattie croniche	Ospedale-ambulatorio; Residenze sanitarie assistenziali (RSA o RSSA); Farmacie; Case di riposo	Portineria, pneumologia, cardiologia, diabetologia...	Medico Infermiere Assistente sanitaria		0-14gg	0-14gg	Tramite contatto telefonico; Tramite email	Tramite contatto telefonico; Tramite email	Si	Assistente sanitaria medico se richiesto da ASI	Si	Si	Si	Percentuale adesione popolazione target invitata; Copertura vaccinale; Costi	Si	Http://www.regione.fvg.it/rafvg/cms/rafvg/salute-sociale/promozione-salute-prevenzione/foglia33/	Si
G	Vaccinazione donne in gravidanza	Gravidanza	Ospedale-ambulatorio	Ginecologie	Medico; Specializzandi di igiene		0-14gg		Tramite contatto telefonico; Segnalazione alla segreteria dell'ambulatorio	Tramite contatto telefonico; Lasciando nome a segreteria ambulatorio	SI	Dirigenti medici /specializzandi in Igiene	SI	SI	SI	Percentuale adesione popolazione target invitata; Gradimento popolazione target; Eventi avversi con chiamata dopo 48/72h	No	Delibera Azienda Policlinco Bari n. 1580 del 02/10/2018 (Processo Operativo per la profilassi vaccinale	No

																		delle donne in gravidanza)	
**H**	Adulti con condizioni a rischio	Diabete	Postazioni vaccinali mobili; Centro diabetologico		Medico; Assistente sanitario o infermiere						Si	Medico presente nel corso dell'iniziativa	Si	No	No				Si
**I**																			
**J**																			
**K**																			
**L**																			
**M**																			
**N**	Anziani di età>65 anni; adulti con condizioni a rischio	Soggetti nefropatici, soggetti con bpco	Ospedale-ambulatorio; residenze sanitarie assistenziali (RSA o RSSA); case di riposo		Medico; infermiere		0-14gg		Con lettera di invito	Con lettera di invito	Si		No	Si	Non so				No
**O**																			
**P**	Anziani di età>65 anni; adulti con condizioni a rischio	Tutti elencati nella circolare	Ospedale-all'interno dei reparti di degenza; ospedale-ambulatorio; case di riposo	Geriatria Servizio pneumologico	Medico infermiere e assistente sanitario	0-14gg	0-14gg	0-14gg			No			Si	Si	Percentuale adesione popolazione target invitata; copertura vaccinale	Si	Https://www.sabes.it/it/dipartimento-prevenzione.asp; https://www.asdaa.it/it/news.asp?aktuelles_action	No
**Q**	Operatori sanitari		Ospedale-all'interno dei reparti di degenza	Tutti i reparti	Medico; infermiere										Non so			Http://www.hproimmune.eu/bulletins/bulletin	No

																		03_eng.pdf	
**R**																			
**S**	Adulti con condizioni a rischio		Ospedale-all'interno dei reparti di degenza; ospedale-ambulatorio; residenze sanitarie assistenziali (RSA o RSSA); case di riposo		Medico; infermiere		0-14gg	0-14gg	Con lettera di invito; tramite email	Con lettera di invito; tramite email	Si	Medico	No	Si	Si	Percentuale adesione popolazione target invitata; copertura vaccinale; costi; gradimento popolazione target	Si		No

STRATEGIA/INIZIATIVA n. 2- VACCINAZIONE ANTINFLUENZALE

	1	2	3	4	5	6.1 [Con prenotazione cartacea]	6.1 [Con prenotazione telefonica]	6.1 [Con prenotazione telematica]	6.2	6.3	7	7.1	7.2	8	9	9.1	9.2	11	12
**A**																			
**B**																			
**C**	Anziani di età>65 anni; Adulti con condizioni a rischio		Postazioni vaccinali mobili; Ospedale-ambulatorio;	Residenze sanitarie assistenziali (RSA o RSSA); Farmacie; Case di reclusione/circondariali;	Medico Infermiere	0-14gg	0-14gg	0-14gg	Con lettera di invito; Tramite sms	Con lettera di invito; Tramite sms	Si	Medico	Si	Si	Si	Percentuale adesione popolazione target invitata; Copertura vaccinale; Costi;	Si		No

				Case di riposo												Gradimento popolazione target			
**D**																			
**E**																			
**F**	Personale sanitario		Ospedale-ambulatorio; distretti sanitari	Servizio prevenzione e protezione	Medico Assistent e sanitaria		0-14gg		Con lettera di invito		Si	Assis tente sanit aria	Si	Si	Si	Percentuale di adesione della popolazione target invitata;<br>copertura vaccinale;<br>costi	Si	http://www.regione.fvg.it/rafvg/cms/RAFVG/salute-sociale/promozione-salute-prevenzione/FOGLIA33/	No
**G**																			
**H**	Operatori Sanitari		Ospedale-all’interno dei reparti di degenza	Ambulatorio messo a disposizione dalla Direzione Medica di Presidio Ospedaliero (generalmente Ambulatorio terapia iniettiva)	Medico;<br>Assistent e Sanitario o Infermier e				Contatto telefonic o con Direzion e Medica di Presidio che avvisa le caposale		N o			N o	N o				No
**I**																			
**J**																			
**K**																			
**L**																			
**M**																			
**N**																			
**O**																			
**P**																			
**Q**																			
**R**																			
**S**																			

STRATEGIA/INIZIATIVA n. 1- VACCINAZIONE ANTI-PNEUMOCOCCICA

	1	2	3	4	5	6.1 [Con prenotazione cartacea]	6.1 [Con prenotazione telefonica]	6.1 [Con prenotazione telematica]	6.2	6.3	7	7.1	7.2	8	9	9.1	9.2	11	12
A	Anziani di età>65 anni; Adulti con condizioni a rischio		Presa in carico												Si				No
B																			
C																			
D	Adulti con condizioni a rischio	Vedi patologie PNPV	Ospedale-all'interno dei reparti di degenza; Ospedale-ambulatorio		Medico; infermiere						Si	Medico infermiere	Si	Si	Si	Copertura vaccinale; Costi	No	UO prevenzione	No
E																			
F																			
G																			
H																			
I																			
J																			
K																			
L																			
M																			
N	Anziani di età>65 anni; Adulti con condizioni a rischio	Pazienti BPCO, nefropatici, cardiopatici, diabetici	Ospedale-ambulatorio; residenze sanitarie assistenziali (RSA o RSSA); case di riposo		Medico; infermiere		0-14 gg		Con lettera di invito; Tramite contatto telefonico	Con lettera di invito; Tramite contatto telefonico	Si	Medicoinfermiere	No	Si	Non so				No
O																			
P	Anziani di età>65 anni		Ospedale-ambulatorio; case di riposo	Geriatria, servizio pneumologico	Medico; infermiere;	0-14 gg	0-14 gg	0-14 gg		Con lettera di invito	No			Si	Si	Percentuale di adesione della popolazione target invitata;	Si	https://www.asdaa.it/it/news.asp?a	No

					assistente sanitario											copertura vaccinale		ktuelles_action; https://www.sabes.it/it/Dipartimento-prevenzione.asp	
**Q**																			
**R**																			
**S**	Adulti con condizioni a rischio		Ospedale-all'interno dei reparti di degenza; ospedale-ambulatorio; residenze sanitarie assistenziali (RSA o RSSA); case di riposo		Medico; infermiere		0-14 gg	0-14 gg	Con lettera di invito; Tramite email	Con lettera di invito; Tramite email	Si	Medico	Si	Si	Si	Percentuale di adesione della popolazione target invitata; copertura vaccinale; costi	Si		No

STRATEGIA/INIZIATIVA n. 1- VACCINAZIONE ANTI-HERPES ZOSTER

	**1**	**2**	**3**	**4**	**5**	**6.1 [Con prenotazione cartacea]**	**6.1 [Con prenotazione telefonica]**	**6.1 [Con prenotazione telematica]**	**6.2**	**6.3**	**7**	**7.1**	**7.2**	**8**	**9**	**9.1**	**9.2**	**11**	**12**
**A**																			
**B**																			
**C**																			
**D**																			
**E**																			
**F**																			
**G**																			
**H**																			
**I**																			
**J**																			
**K**																			
**L**																			
**M**																			
**N**	Anziani di età>65 anni; Adulti con condizioni a rischio	Dializzati	Ospedale-ambulatorio		Medico; infermiere	0-14 gg	0-14 gg		Tramite contatto telefonico	Tramite contatto telefonico	Si	Medico/infermiere	No	Si	Non so				No
**O**																			
**P**																			
**Q**																			
**R**																			

S																			
